# Venetoclax resistance induced by activated T cells can be counteracted by sphingosine kinase inhibitors in chronic lymphocytic leukemia

**DOI:** 10.3389/fonc.2023.1143881

**Published:** 2023-03-20

**Authors:** Valeria J. Sarapura Martinez, Brenda Buonincontro, Chiara Cassarino, Juliana Bernatowiez, Ana Colado, Gregorio Cordini, Maria del Rosario Custidiano, Carolina Mahuad, Miguel A. Pavlovsky, Raimundo F. Bezares, Nicolás O. Favale, Mónica Vermeulen, Mercedes Borge, Mirta Giordano, Romina Gamberale

**Affiliations:** ^1^ Laboratorio de Inmunología Oncológica, Instituto de Medicina Experimental (IMEX)-Consejo Nacional de Investigaciones Científicas y Técnicas (CONICET)- Academia Nacional de Medicina (ANM), Buenos Aires, Argentina; ^2^ Servicio de Hematología, Hospital de Clínicas, José de San Martín, Universidad de Buenos Aires (UBA), Buenos Aires, Argentina; ^3^ Departamento de Hematología y Unidad de Trasplante Hematopoyético, Instituto Alexander Fleming, Buenos Aires, Argentina; ^4^ Servicio de Hematología, Hospital Alemán, Buenos Aires, Argentina; ^5^ Hematología, FUNDALEU, Buenos Aires, Argentina; ^6^ Servicio de Hematología, Hospital Teodoro Álvarez, Buenos Aires, Argentina; ^7^ Cátedra de Biología Celular y Molecular, Facultad de Farmacia y Bioquímica, Universidad de Buenos Aires (UBA), Buenos Aires, Argentina; ^8^ Instituto de Química y Fisicoquímica Biológicas “Profesor Dr. Alejandro C. Paladini” (IQUIFIB), Consejo Nacional de Investigaciones Científicas y Técnicas (CONICET) - Universidad de Buenos Aires, Buenos Aires, Argentina; ^9^ Laboratorio de Células Presentadoras de Antígeno y Respuesta Inflamatoria, IMEX-CONICET-ANM, Buenos Aires, Argentina; ^10^ Departamento de Microbiología, Parasitología e Inmunología, Facultad de Medicina, UBA, Buenos Aires, Argentina

**Keywords:** sphingosine kinases, activated T cells, Bcl-XL, CLL (chronic lymphocytic leukemia), venetoclax resistance, SKI-II, opaganib

## Abstract

The treatment of chronic lymphocytic leukemia (CLL) patients with venetoclax-based regimens has demonstrated efficacy and a safety profile, but the emergence of resistant cells and disease progression is a current complication. Therapeutic target of sphingosine kinases (SPHK) 1 and 2 has opened new opportunities in the treatment combinations of cancer patients. We previously reported that the dual SPHK1/2 inhibitor, SKI-II enhanced the *in vitro* cell death triggered by fludarabine, bendamustine or ibrutinib and reduced the activation and proliferation of chronic lymphocytic leukemia (CLL) cells. Since we previously showed that autologous activated T cells from CLL patients favor the activation of CLL cells and the generation of venetoclax resistance due to the upregulation of BCL-XL and MCL-1, we here aim to determine whether SPHK inhibitors affect this process. To this aim we employed the dual SPHK1/2 inhibitor SKI-II and opaganib, a SPHK2 inhibitor that is being studied in clinical trials. We found that SPHK inhibitors reduce the activation of CLL cells and the generation of venetoclax resistance induced by activated T cells mainly due to a reduced upregulation of BCL-XL. We also found that SPHK2 expression was enhanced in CLL cells by activated T cells of the same patient and the presence of venetoclax selects resistant cells with high levels of SPHK2. Of note, SPHK inhibitors were able to re-sensitize already resistant CLL cells to a second venetoclax treatment. Our results highlight the therapeutic potential of SPHK inhibitors in combination with venetoclax as a promising treatment option for the patients.

## Introduction

Leukemic B cells from chronic lymphocytic leukemia (CLL) patients circulate between peripheral blood and lymphoid tissues where they survive, become activated, and proliferate in close contact with activated T cells, myeloid cells and by receiving signals through the B-cell receptor (BCR). In the last years, the approval of multiple new targeted therapy drugs that affect the proliferation or survival of CLL cells, improved both clinical outcomes and quality of life of CLL patients. One of them is venetoclax (1), a potent and selective inhibitor of the antiapoptotic protein BCL-2, that binds specifically to the hydrophobic groove of BCL-2, displacing proapoptotic proteins and rapidly inducing apoptosis in cells that rely on BCL-2 for survival (2). It has demonstrated efficacy and a safety profile in CLL and other hematological malignancies, both in clinical trials (1, 3, 4) and in real world setting (5). While CLL patients treated with venetoclax can reach deep clinical responses, particularly when used in combination with an anti-CD20 monoclonal antibody, and/or inhibitors of Bruton tyrosine kinase (3, 4, 6), the emergence of resistant cells is a current complication. Multiple independent mechanisms contributed to venetoclax resistance, including the acquisition of various mutations in BCL-2 and/or the upregulation of other anti-apoptotic proteins which are not targeted by venetoclax, such as BCL-XL and MCL-1 (7, 8). Different groups reported that malignant cells that recently interacted *in vivo* with the supportive microenvironment of lymphoid tissues (9, 10), or those that were cultured *in vitro* with different signals that mimic microenvironment stimuli (11–15), show an increased expression of BCL-XL and MCL-1 and are less sensitive to venetoclax compared to quiescent or unstimulated CLL cells. In line with this, we previously reported that when peripheral blood mononuclear cells (PBMC) from CLL patients were incubated on immobilized anti-CD3 monoclonal antibodies (aCD3) to induce T cell activation, autologous activated T lymphocytes induced the activation of CLL cells, and *in vitro* venetoclax resistance due to the upregulation of BCL-XL and MCL-1 (11, 12). Moreover, we found that venetoclax-resistant CLL cells show a highly activated and proliferative phenotype and a sustained resistance to a second treatment with the drug (11).

Sphingosine kinases (SPHK) participate in the regulation of bioactive sphingolipid metabolism and mediate several biological functions, including cell growth, differentiation, survival and migration, among others (16). SPHK has two isoforms, SPHK1 and SPHK2, which mediate the phosphorylation of sphingosine to form sphingosine 1-phosphate (S1P) (17). Therapeutic targeting of SPHK has attracted enormous attention and opened new opportunities in the treatment combinations of cancer patients. In line with this, our previous work on SHPK in CLL confirmed that SKI-II, the most well-characterized dual SPHK1/2 inhibitor (18), induced CLL cell death in a dose-dependent way (19). Moreover, non-apoptotic doses of SKI-II enhanced the cell death triggered by fludarabine, bendamustine or the targeted drug ibrutinib which inhibit Bruton’s tyrosine kinase (BTK) and IL-2–inducible T cell kinase (ITK) (19). Interestingly, sub-apoptotic doses of SKI-II also reduced the activation and proliferation of CLL cells induced by different signals that mimic the tumor microenvironment, including anti-IgM plus CD40L (19). In addition, others found that safingol, a known SPHK1 inhibitor which was recently found to be a substrate for SPHK2 (20), potentiates the anti-cancer effect of a botanical drug called Polyphenon E™ (21). In solid tumors, opaganib, an orally active, isozyme-selective inhibitor of SPHK2 with antitumor and anti-inflammatory activity, which can be safely administered to severely compromised patients with solid and hematological tumors or Covid-19 (22), restores the sensitivity of BRAFV600E mutant colon cancer cells to vemurafenib (23) and it has being studied in clinical trials with patients with metastatic castration-resistant prostate cancer progressing on abiraterone or enzalutamide (NCT04207255) and with advanced cholangiocarcinoma patients in combination with hydroxychloroquine sulfate (NCT03377179). The combination of SPHK inhibitors and venetoclax in CLL cells was not evaluated yet.

To further study the role of SPHK in CLL, we herein determine whether SPHK inhibitors can reduce the emergence of *in vitro* venetoclax resistance in CLL cells and/or are able to induce the cell death of already venetoclax-resistant cells. Our results suggest that a combined therapy of venetoclax and SPHK inhibitors may be a promising treatment option for CLL patients in the future.

## Methods

Peripheral blood samples were collected from twenty-eight unrelated CLL patients. All samples used in this study were obtained after informed consent in accordance with the Declaration of Helsinki and with Institutional Review Board approval from the Academia Nacional de Medicina, Buenos Aires, Argentina. CLL was diagnosed according to standard clinical and laboratory criteria. At the time of analysis, all patients were free from clinically relevant infectious complications and were treatment naïve or had not received treatment for ≥3 months before the investigation began. The main clinical and biological characteristics of the patients enrolled in our study are summarized in **Supplementary Table 1**.

In the present study we employed 0,2 µM of venetoclax, a concentration achievable *in vivo* since 1 μM of venetoclax can be found in plasma of treated CLL patients in the steady state (1, 24). This concentration was selected because in our previous study we demonstrated that autologous activated T cells promote CLL resistance to doses of venetoclax ranged between 0.01 and 1 uM (11, 12). In the case of SPHK inhibitors, opaganib was employed at 15 µM because up to 13,5 µM (25) or 16 µM (26) can be found in plasma of treated patients. SKI-II was not evaluated in clinical trials yet, but its concentration of 15 µM was selected based on our previous *in vitro* studies (19). A detailed description of materials and methods employed in this study can be found in the online **Supplementary Data**.

## Results

### Venetoclax-resistant CLL cells express high levels of SPHK2

**Figure 1:**
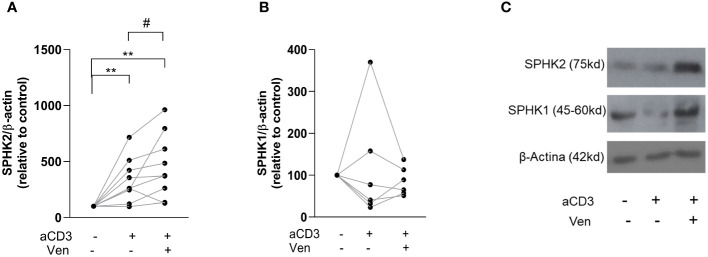
Venetoclax resistant CLL cells show high expression of SPHK2. PBMC from CLL patients (4x10^6^ cells/ml) were cultured in complete medium with aCD3 or the isotype control. After 48 h, venetoclax (Ven) 0,2 µM or DMSO were added to the cultures. After 24 h, non-viable cells were excluded by employing a dead cell removal kit and then CLL cells were purified using a CLL purification kit as detailed in the **Supplementary Material** and methods section. Then, whole cell lysates were prepared with purified viable CLL cells and proteins were separated on a standard 12% SDS-PAGE and transferred to a PVDF membrane. Membranes were probed with primary antibodies for SPHK1, SPHK2 and β-Actin, followed by the corresponding secondary antibody. Then, specific bands were quantified by employing ImageJ and quantitative densitometry protein expression relative to β-actin as loading control was obtained for each culture condition. The figure shows the expression of SPHK2 (n=9) **(A)** and SPHK1 (n=6) **(B)** relative to the expression obtained in control cultures. To compare aCD3 and aCD3+Ven versus control cultures we employed Wilcoxon Signed Rank Test, **p < 0.01. The comparison between aCD3 and aCD3+Ven was performed using Wilcoxon matched-pairs signed rank test, # p < 0.05. **(C)** The figure shows the results obtained with one representative CLL sample.

When we studied the phenotype of venetoclax-resistant CLL cells induced by activated T cells, we found that they showed an aggressive phenotype characterized by higher CD86, PD-1, Ki-67 and MCL-1 and/or BCL-XL expression with sustained resistance to a second treatment with the drug (11, 12). Since we previously reported that SPHKs regulate CLL cell survival, activation and proliferation (19), we hypothesized that venetoclax-resistant cells would also express high levels of SPHKs. To test this hypothesis, venetoclax resistant cells were generated as previously described (11, 12) by culturing PBMC from CLL patients with or without aCD3 for 72 h in the presence of DMSO or venetoclax during the last 24 h of culture. Then, SPHK1 and SPHK2 were evaluated by western blot in viable purified CLL cells. Because most of CLL cells die in control cultures with venetoclax, this condition was not assessed. As it is shown in **Figure 1A**, the presence of autologous activated T cells enhanced SPHK2 expression in CLL cells, which was higher in those resistant to venetoclax. SPHK1 expression was not consistently modified under these culture conditions (**Figure 1B**). Non*-*normalized data of the expression of SPHK1 and 2 are shown in **Supplementary Figure 1**. Results obtained with a representative CLL patient are shown in **Figure 1C**.

### SPHK inhibitors reduce the generation of venetoclax resistance induced by activated T cells

**Figure 2:**
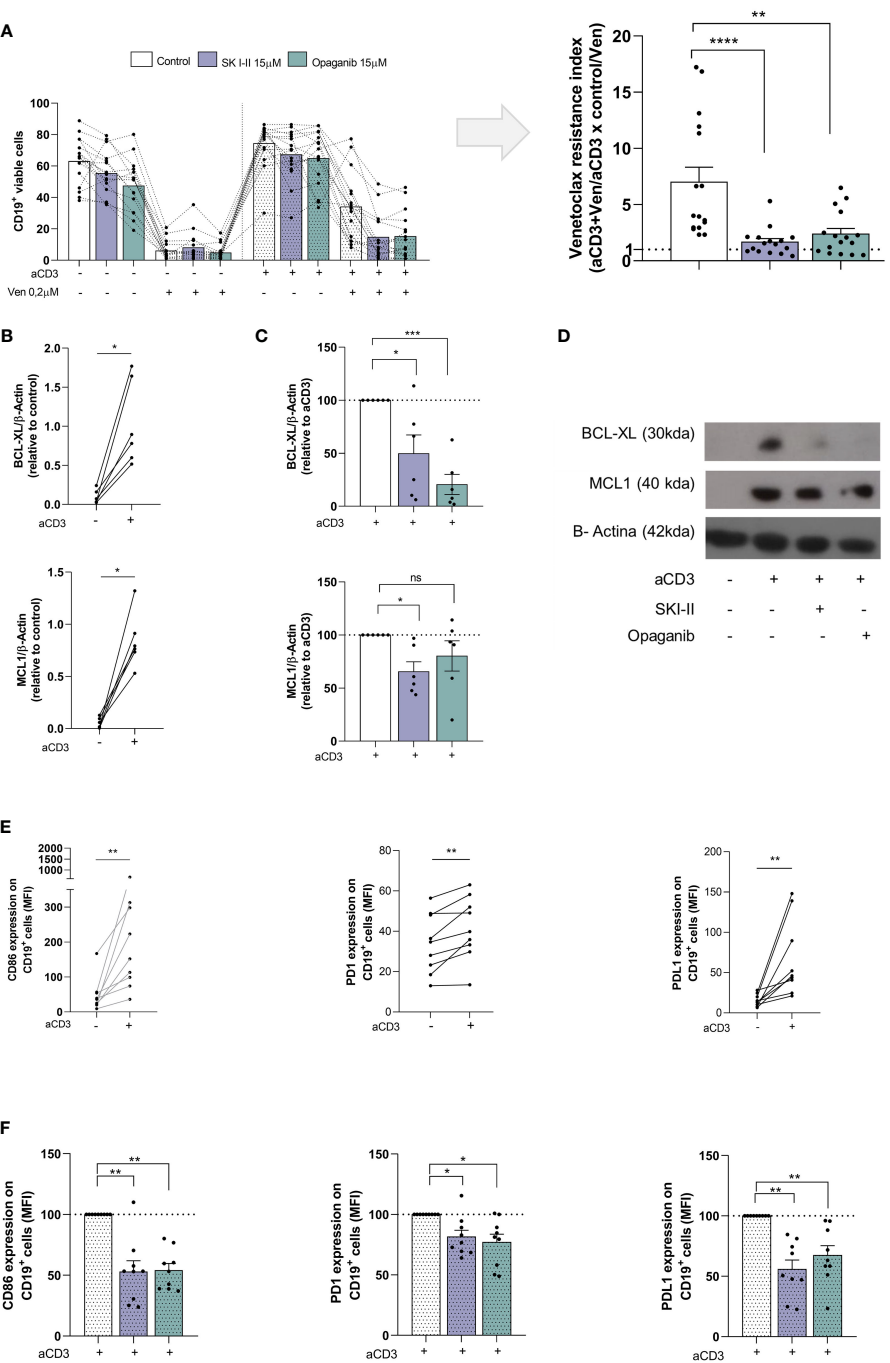
SKI-II and opaganib prevent venetoclax resistance and CLL activation induced by autologous activated T cells. PBMC from CLL patients (4x10^6^ cells/ml) were cultured in complete medium with aCD3 or the isotype control in presence or absence of SKI-II 15 µM and opaganib 15 µM for 48 h. Then, venetoclax (Ven) 0,2 µM or DMSO were added to the cultures for additional 24 h. **(A)** CD19^+^ cell survival was evaluated by flow cytometry as detailed in **Supplementary Methods**. Left panel: The figure shows the mean ± SEM of CD19^+^ cell survival in each condition. Right panel: With the values of CD19^+^ cell survival obtained in control, venetoclax (Ven), aCD3 and aCD3+Ven cultures we calculated the venetoclax resistance index for each patient as follows: (aCD3+Ven/aCD3) x (control/Ven). A value higher than 1 indicates that aCD3 cultures favor venetoclax resistance. The figure shows the mean ± SEM of venetoclax resistance index. Statistical analysis was performed using Friedman test followed by Dunn´s test, ** p<0.01, **** p<0.0001 (n=16). **(B-D)** Purified CLL cells from control and aCD3 cultures with and without SKI-II and opaganib at 48 h were analyzed by western blot as detailed in **Figure 1**, but in this case, membranes were probed with primary antibodies for BCL-XL, MCL-1 and β-Actin, followed by the corresponding secondary antibody. **(B)** The figures show the expression of MCL-1 and BCL-XL on leukemic cells from control and aCD3 cultures. Statistical analysis was performed using Wilcoxon matched-pairs signed rank test, * p<0.05, NS stands for Not Statistically Significant (n=6). **(C)** The figures show the expression of MCL-1 and BCL-XL in leukemic cells from aCD3+SKI-II and aCD3+opaganib cultures relative to the expression obtained in aCD3 cultures. To compare aCD3+SKI-II and aCD3+opaganib versus aCD3 cultures we employed One sample t test, * p<0.05, ***p<0.001 (n=6) **(D)** The figure shows the results obtained with one representative CLL sample (CLL #23 in **Supplementary table 1**). **(E, F)** PBMC from CLL patients were cultured with aCD3 or the isotype control in presence or absence of SKI-II 15 µM and opaganib 15 µM for 48 h. The expression of CD86, PD1 and PDL1 on CD19^+^ cells was evaluated by flow cytometry. **(E)** The figures show the CD86, PD1 and PDL1 expression on CD19^+^ cells in control and aCD3 cultures. Statistical analysis was performed using Wilcoxon matched-pairs signed rank test, ** p<0.01 (n=9). **(F)** The figure shows the mean ± SEM of the expression of CD86, PD1 and PDL1 on CD19^+^ cells in aCD3 cultures with SKI-II and opaganib relative to aCD3 cultures without SPHK inhibitors. Statistical analysis was performed using Wilcoxon Signed Rank Test, * p<0.05, ** p<0.01 (n=9).

The results mentioned above prompted us to evaluate whether SPHK inhibitors affect the generation of venetoclax resistance. To this aim, we employed the dual SPHK1/2 inhibitor SKI-II and the SPHK2 inhibitor opaganib. PBMC from CLL patients were cultured with or without aCD3 in the presence of DMSO, SKI-II (15 µM) or opaganib (15 µM) for 72 h, with the addition of DMSO or venetoclax during the last 24 h. The survival of CLL cells were evaluated by flow cytometry. The percentages of viable CLL cells in each culture condition are shown in **Figure 2A** (left panel). SPHK inhibitors did not significantly modify the survival of CLL cells in control cultures or aCD3 cultures with DMSO. As we previously reported, CLL cells from aCD3 cultures were less sensitive to venetoclax treatment compared to CLL cells from control cultures. Of note, in the presence of SKI-II or opaganib leukemic cells in aCD3 cultures with venetoclax showed reduced survival values compared to CLL cells in this culture condition without SPHK inhibitors. To easily show the effect of SPHK inhibitors on this process we calculated the venetoclax resistance index for each patient (**Figure 2A**, right panel). Resistance indexes compare venetoclax-induced cell death in control and in aCD3 cultures and were calculated with the survival values shown in the left panel of **Figure 2A**, as we previously reported (12). Values near one show that CLL cells from control and aCD3 cultures similarly die in response to venetoclax treatment, while higher values indicate that aCD3 cultures favor venetoclax resistance. As it is shown in the right panel of **Figure 2A**, both SPHK inhibitors significantly reduce venetoclax resistance indexes. Dot plots showing leukemic cell survival in a representative experiment are shown in **Supplementary Figure 2**. The heterogeneous venetoclax resistance indexes observed in **Figure 2A** could not be associated with the clinical stage of the patients, their mutational status, or the expression of CD38 and CD49d on leukemic cells (**Supplementary Figure 3**).

### SPHK inhibitors reduce the upregulation of BCL-XL and MCL-1 and the activation of CLL cells induced by activated T cells

Given that aCD3 cultures favored venetoclax resistance by the upregulation of BCL-XL and MCL-1 in CLL cells (11, 12), we wondered whether SPHK inhibitors are able to impair the upregulation of these proteins. To this aim, PBMC from CLL patients were cultured with or without aCD3 in the presence of DMSO, SKI-II and opaganib for 48 h and BCL-XL and MCL-1 expression was evaluated by western blot in purified viable CLL cells. We confirmed that CLL cells in aCD3 cultures expressed high levels of BCL-XL and MCL-1 (**Figure 2B**). As it is shown in **Figure 2C** both SPHK inhibitors reduced the upregulation of BCL-XL on malignant cells induced by activated T cells while MCL-1 expression was only reduced by SKI-II under these culture conditions. The results obtained with a representative CLL patient are shown in **Figure 2D**. We also confirmed that CLL cells in aCD3 cultures became activated (11, 12, 27), and favored the upregulation of CD86, PD-1 and PD1-L on CLL cells (**Figure 2E**) which was impaired by SKI-II and opaganib (**Figure 2F**). Non*-*normalized data of **Figures 2C** and **F** are shown in **Supplementary Figure 4**.

### SPHK inhibitors slightly reduce CD4^+^ and CD8^+^ T cell activation without affecting their survival

**Figure 3:**
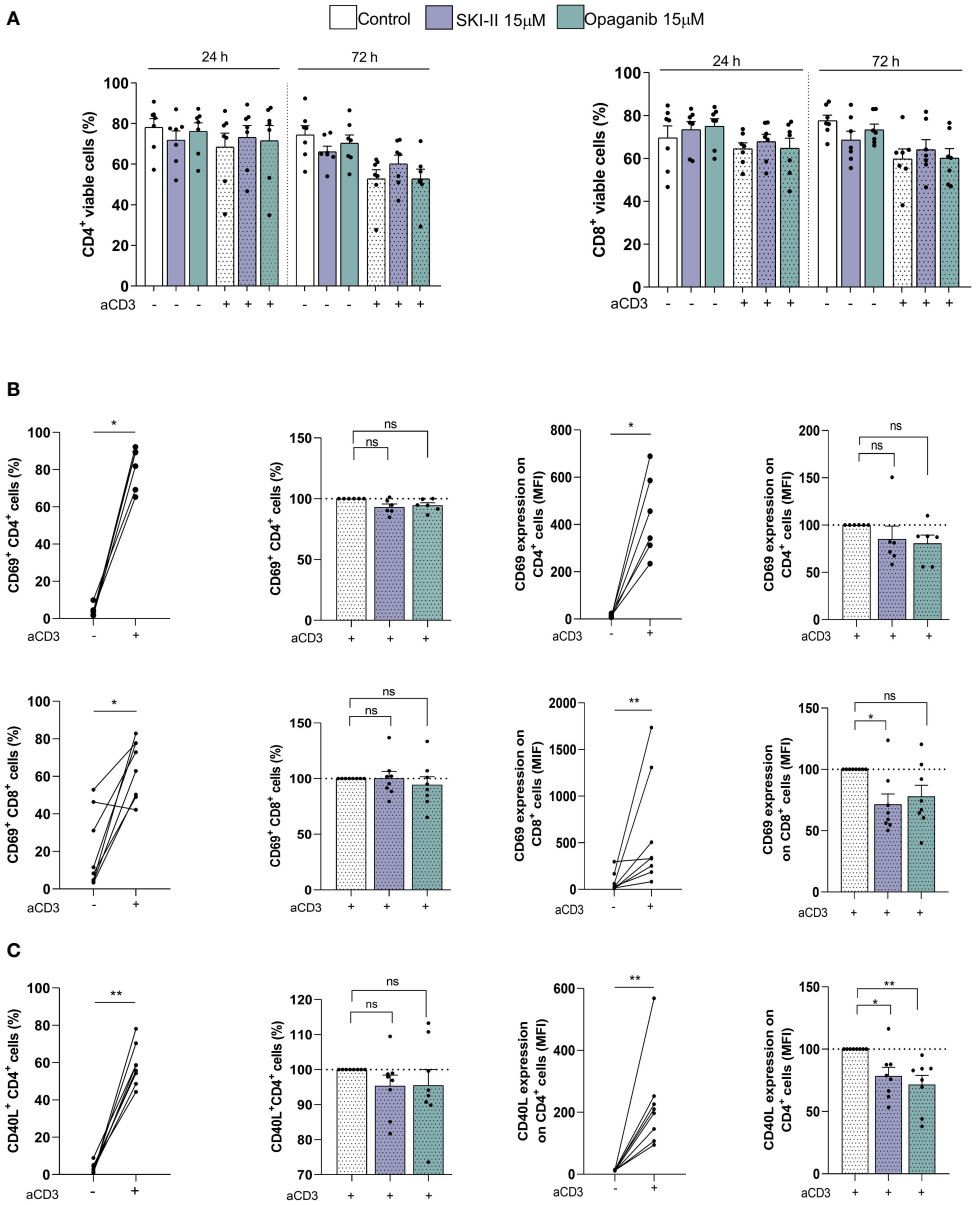
SPHK inhibitors slightly reduce CD4^+^ and CD8^+^ T cell activation without affecting their survival. PBMC from CLL patients (4x10^6^ cells/ml) were cultured in complete medium with aCD3 or the isotype control in presence or absence of SKI-II 15 µM and opaganib 15 µM for 72 h. **(A)** CD4^+^ and CD8^+^ cell survival was evaluated by flow cytometry as detailed in **Supplementary Methods** at 24 and 72 h. The figure shows the mean ± SEM of CD4^+^ (left panel) and CD8^+^ (right panel) cell survival in each condition. Statistical analysis to compare the survival of the cells with or without SPHK inhibitors was performed using Friedman test (n=7). **(B)** The expression of CD69 on CD4^+^ (n=6) and CD8^+^ cells (n=8) was evaluated by flow cytometry at 24 h as detailed in **Supplementary Methods**. Left panel: the figures show the percentage of CD69^+^ CD4^+^ and CD69^+^ CD8^+^ cells in control and aCD3 cultures, and in aCD3 cultures with SKI-II and opaganib relative to aCD3 cultures without SPHK inhibitors. Statistical analysis was performed using Wilcoxon test and Wilcoxon matched-pairs signed rank test, * p<0.05. Right panel: the figures show the mean ± SEM of CD69 expression on CD4^+^ and CD69^+^ CD8^+^ cells in control and aCD3 cultures, and in aCD3 cultures with SKI-II and opaganib relative to aCD3 cultures without SPHK inhibitors. Statistical analysis was performed using Wilcoxon test and Wilcoxon matched-pairs signed rank test, * p<0.05. **(C)** The expression of CD40L on CD4^+^ cells was evaluated by flow cytometry at 24 h as detailed in **Supplementary Methods**. The figure shows the mean ± SEM of the percentage of CD40L^+^ CD4^+^ cells and the expression of CD40L on CD4^+^ cells in control and aCD3 cultures and in aCD3 cultures with SKI-II and opaganib relative to aCD3 cultures without SPHK inhibitors at 24 h. Statistical analysis was performed using Wilcoxon Signed Rank Test and Wilcoxon matched-pairs signed rank test, * p<0.05, ** p<0.01 (n=8). NS stands for Not Statistically Significant.

Since the activation of T lymphocytes favors the generation of CLL cells that are less sensitive to venetoclax, the strong reduction in venetoclax resistance due to SPHK inhibitors (**Figure 2A**) might involve an effect on T cell activation and/or survival. To test this hypothesis, PBMC from CLL patients were incubated with or without aCD3 in the presence of DMSO, SKI-II or opaganib for 72 h and the survival of CD4^+^ and CD8^+^ T cells was evaluated by flow cytometry. As it is shown in **Figure 3A** SPHK inhibitors did not significantly affect the survival of CD4^+^ and CD8^+^ T lymphocytes at the times evaluated. On the other hand, we confirmed that both CD4^+^ and CD8^+^ lymphocytes were activated upon CD3 crosslinking since they upregulated the activation marker CD69 and increased the percentage of CD69^+^ cells at 24 h (**Figure 3B**). SPHK inhibitors did not modify the expression of CD69 on CD4^+^ cells, while SKI-II only slightly reduced the level of CD69 expression on CD8^+^ cells (**Figure 3B**). When the expression of CD40L was evaluated, both SPHK inhibitors only slightly reduced the expression of CD40L induced on CD4^+^ cells upon CD3 crosslinking (**Figure 3C**). Non*-*normalized data of **Figures 3B, C** are shown in **Supplementary Figure 5**.

### SPHK inhibitors induce the cell death of venetoclax-resistant cells

**Figure 4:**
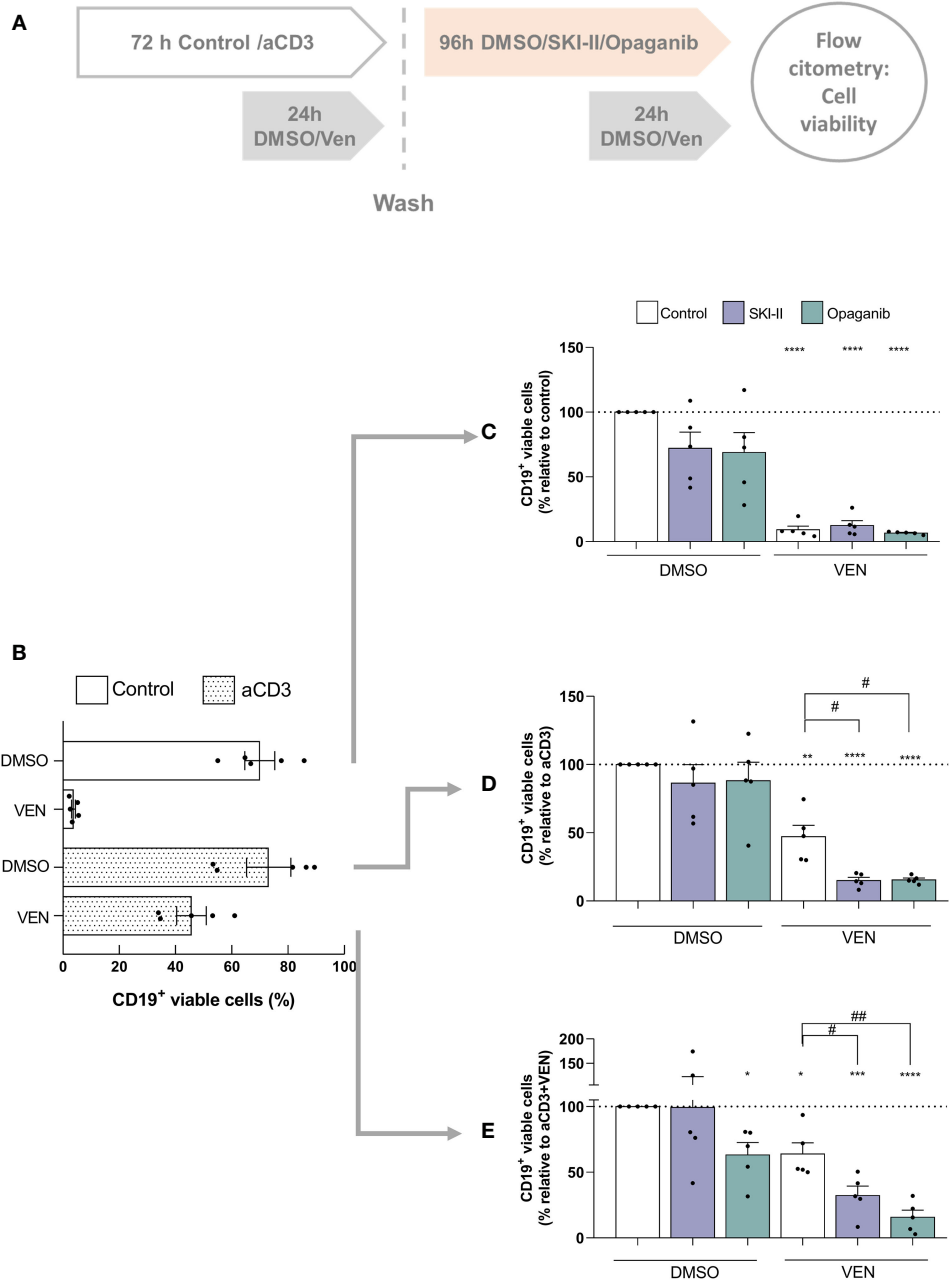
SPHK inhibitors re-sensitize resistant CLL cells to a second venetoclax treatment. **(A)** Schematic diagram of the culture protocol. **(B)** PBMC from CLL patients (4x10^6^ cells/ml) were cultured in complete medium with aCD3 or the isotype control for 72 h in the presence of DMSO or venetoclax (Ven) during the last 24 h of culture. The figure shows the mean ± SEM of CD19^+^ cell survival in each condition. PBMC from control cultures **(C)**, aCD3 cultures **(D)** and aCD3+VEN cultures **(E)** were washed and cultured with DMSO, SKI-II 15 µM or opaganib 15 µM for 96 h combined with DMSO or venetoclax (Ven) during the last 24 h of culture. Statistical analysis was performed using One sample t test. **** p< 0.0001 (n=5) *** p< 0.001 ** p< 0.01 *p< 0.05 (n=5). RM one-way ANOVA, followed by Holm-Sidak’s multiple comparisons test, # p< 0.05 ##p< 0.01 (n= 5).

Finally, given that venetoclax resistant cells express high levels of SPHK2 (**Figure 1A**) we aimed to determine whether SPHK inhibitors can affect the survival of already venetoclax resistant cells. To this aim, PBMC from CLL patients were cultured as mentioned above, alone (control cultures) or with aCD3 for 72 h (aCD3 cultures) in the presence of DMSO or venetoclax during the last 24 h. After that, cells from control cultures with DMSO, aCD3+DMSO and aCD3+VEN were washed and cultured again with DMSO, SKI-II or opaganib for another 96 h, combined with DMSO or venetoclax during the last 24 h of culture as detailed in **Figure 4A**. Because most of CLL cells die in control cultures with venetoclax (**Figure 4B**), this condition was not assessed.

As expected, CLL cells that came from control cultures rapidly died in response to venetoclax and were not significantly affected by the presence of SPHK inhibitors (**Figure 4C**). Similarly, SPHK inhibitors alone did not affect the survival of CLL cells that came from aCD3+DMSO cultures but promoted the cell death induced by venetoclax (**Figure 4D**). Of note, when venetoclax resistant CLL cells that came from aCD3+venetoclax cultures were evaluated we found that, despite the aggressive phenotype acquired under this culture condition (11), they died in response to opaganib alone showing a 45% of cell death (**Figure 4E**). Moreover, even though 64% of already venetoclax resistant cells were able to survive to a second drug exposure, 50% of these cells died due to the presence of venetoclax in combination with SKI-II and 75% in combination with opaganib, showing that SPHK inhibitors were able to re-sensitize resistant CLL cells to a second venetoclax treatment (**Figure 4E**). Non*-*normalized data of **Figures 4C–E** are shown in **Supplementary Figure 6**.

## Discussion

The present study seeks to address the role of SPHK inhibitors in the context of venetoclax resistance induced in CLL cells by autologous activated T lymphocytes. Within lymphoid tissues and particularly in lymph nodes, CLL cells obtain a distinctly activated gene signature in comparison with the peripheral blood due to the array of signals from the tumor microenvironment that favor not only their activation, but also their survival and proliferation (28, 29). T cells were shown to be an important source of the stimuli that CLL cells receive within lymph nodes (30). In line with this, we here confirmed that *in vitro* activation of autologous T cells induces the activation of CLL cells and the emergence of venetoclax resistance (11, 12, 27). To determine whether SHPK inhibitors can reduce the generation of venetoclax resistance in our *in vitro* system, we employed SKI-II, which inhibits both isoforms of SHPK, and opaganib, a SPHK2 inhibitor that is currently in Phase 2 clinical testing in patients having cholangiocarcinoma (NCT03377179) or prostate cancer (NCT04207255). The fact that both inhibitors reduced the generation of venetoclax-resistant CLL cells induced by autologous activated T lymphocytes suggests that the inhibition of SHPK2 may be involved in this process.

In an attempt to understand how SPHK inhibitors reduce the generation of venetoclax resistance in our *in vitro* system, we evaluated the expression of BCL-XL and MCL-1 in CLL cells, which are not targeted by venetoclax. Given that activated T cells induced the upregulation of these molecules in CLL cells (11, 12), we hypothesized that SPHK inhibitors would impair their expression. When the upregulation of BCL-XL induced in CLL cells by autologous activated T cells was evaluated, we found that both SPHK inhibitors reduced it, suggesting that SPHK2 may participate in this process. On the other hand, when MCL-1 was evaluated, we found that its upregulation induced by activated T cells was not consistently affected by opaganib and only reduced in a 40% by the presence of SKI-II, suggesting that the activation of SPHK1 might be involved, at least in part, in the upregulation of MCL-1 in CLL cells. The role of SPHK1 and 2 in MCL-1 expression on malignant cells seems to differ depending on the cell type. Thus, inhibition of SPHK1 in human acute myeloid leukemia cells, but not the inhibition of SPHK2, induces MCL-1 degradation (31). Similarly, SPHK1 inhibition reduce BCR/ABL-induced upregulation of MCL-1 in chronic myeloid leukemia cells (32). On the contrary, in large granular lymphocyte leukemia (33) or multiple myeloma (34) SPHK2 inhibition downregulates MCL-1 expression.

Based on our results, it seems that the reduction in venetoclax resistance exerted by SKI-II and opaganib might principally rely on the impairment of BCL-XL upregulation. The role of MCL-1 and BCL-XL in the resistance to venetoclax is still a matter of debate. While Liu et al. reported that the increase in MCL-1 expression is one important mechanisms for venetoclax resistance (35), others found that BCL-XL is a major regulator of this process in CLL (9, 36). In line with our result, Haselager et al. demonstrated that there is a hierarchy of BCL-2 family members in CLL cells under the pressure of venetoclax, in which BCL-XL is dominant over MCL-1 in CLL venetoclax resistance when both are present (9, 36).

Regarding CLL activation induced by activated T cells, the fact that both SPHK inhibitors similarly reduced the upregulation of the activation markers CD86, PD-1 and PDL1 induced by the presence of activated T lymphocytes, suggests that SPHK2 might participate in the upregulation of these molecules. However, since in our *in vitro* assay, PBMC from CLL patients were cultured on aCD3 to induce T cell activation and the presence of activated T lymphocytes favor the activation of the leukemic clone, the reduced CLL activation that SKI-II and opaganib induced in our system, may involve an effect on the T cell compartment. When we evaluated the effect of SPHK inhibitors on the survival and activation of CD4^+^ and CD8^+^ T cells from CLL patients, we found that SKI-II and opaganib slightly reduce T cell activation, without affecting their survival. Given that in murine T cells SPHK inhibition and/or ablation improves T cell mediated tumor control against murine melanoma and increases the secretion of an array of cytokines in response to stimulation, further experiments are warranted to evaluate whether this is also the case in T cells of CLL patients (37–39). In summary, the reduction in CLL activation exerted by SPHK inhibitors in our system may involve a direct effect on CLL cells and indirect effects on other subpopulations present in PBMC cultures, including CD4^+^ and CD8^+^ T cells.

We here found that SPHK2 expression was clearly enhanced by the presence of activated T cells of the same patient and the presence of venetoclax selects resistant cells with high levels of SPHK2. This observation encouraged us to test whether SPHK2 inhibition favor the cell death of these already resistant cells, so we first generated venetoclax resistant cells and then cultured them with SPHK inhibitors in combination with venetoclax. Of note, despite the aggressive phenotype of venetoclax-resistant cells (11) these cells die when were cultured again with a second venetoclax treatment in combination with SKI-II and opaganib (**Figure 4E**). We are aware that our observations are limited to an *in vitro* venetoclax resistant assay. However, when we analyzed a public data set of CLL cells from 7 patients with persistent MRD after 1 year on venetoclax-based therapy (RNA-Seq GSE192685 (40)), we found that 5 out of 7 CLL samples showed higher SPHK2 expression at progression on venetoclax therapy (SC2) compared to the values prior to venetoclax treatment (SC1) (**Supplementary 7**). It remains to be determined whether co-treatment with SPHK inhibitors could re-sensitize these *in vivo* venetoclax resistant CLL cells.

Despite the efficacy of venetoclax based regimens, minimal residual disease recrudescence and progressive disease are common with extended follow-up (41). The failure of therapeutic regimens to eradicate malignant cells often results from the outgrowth of minor subclones with more dangerous and aggressive phenotype. In line with this, it was already reported that an intraclonal complexity exists in CLL (42). Thus, the leukemic clone contains a spectrum of cells from the “proliferative fraction” to the “quiescent fraction”. While the first one is enriched in recently born/divided cells mostly present in lymphoid tissues that can be also found in peripheral blood as a small proportion of recently emigrant CLL cells, the “quiescent fraction” enriched in older, less vital cells, are mostly present in peripheral blood and need to immigrate to lymphoid tissue or die (42). Two independent groups recently demonstrated that in treatment naïve patients the small proportion of recently emigrant CLL cells overexpress anti-apoptotic proteins including MCL-1 and BCL-XL (9, 10). Of note, this subpopulation survives and increases upon *in vivo* venetoclax treatment of the patients showing that resistant CLL cells already exist, even in untreated CLL patients and these cells persist during proapoptotic treatment with venetoclax (10). Further studies are warranted in order to determine whether in treatment naïve patients the small proportion of recently emigrant CLL cells that overexpress MCL-1 and BCL-XL also express high levels of SPHK2.

As we already mentioned above, the fact that SKI-II and opaganib similarly diminish the generation of venetoclax-resistance and re-sensitize already venetoclax-resistant CLL cells to the drug suggest that the inhibition of SHPK2 is involved in this process. However, while opaganib is a very well-known SPHK2 inhibitor that reduces sphingosine-1 phosphate levels, it may also inhibit other enzymes in the sphingolipid metabolism. Thus, by inhibiting dihydroceramide desaturase opaganib increases dihydroceramides, and by targeting glucosylceramide synthase reduces hexosylceramides (22). Since glucosylceramide synthase inhibitors were reported to sensitize CLL cells to chlorambucil and fludarabine induced cell death (43), further experiments are needed to confirm whether SKI-II and opaganib effects on CLL cells are meditated by the inhibition of SHPK2 and/or other molecule(s).

In conclusion, our results highlight the therapeutic potential of SPHK inhibitors in combination with venetoclax as a promising treatment option for the patients. Undoubtedly, comparative clinical studies are needed to clearly demonstrate whether SPHK inhibitors are good partners of venetoclax in CLL.

## Data availability statement

The original contributions presented in the study are included in the article/**Supplementary Material**. Further inquiries can be directed to the corresponding author.

## Ethics statement

The studies involving human participants were reviewed and approved by Institutional Ethics Committee from the Institutes of the Academia Nacional de Medicina, Buenos Aires, Argentina. The patients/participants provided their written informed consent to participate in this study.

## Author contributions

VS did most of the experiments and created the Figures. BB, CC, JB, AC, and MV contributed in the purification of CLL samples, in CD38 and CD49d determination by flow cytometry and in the analysis, interpretation and discussion of the data. GC, MC, CM, MP, and RB provided patients samples and advice and contributed in the analysis and interpretation of the data; NOF contributed in the analysis, interpretation and discussion of the data; MG and MB participated in the project conception and critically reviewed the manuscript, which was written by VS and RG. RG designed and supervised the study. All authors approved the final version of the manuscript.
