# Supplementary material for: Venetoclax resistance induced by activated T cells can be counteracted by sphingosine kinase inhibitors in chronic lymphocytic leukemia
Source: Front Oncol. 2023 Mar 20;13:1143881. doi: 10.3389/fonc.2023.1143881 (PMC10067719; doi:10.3389/fonc.2023.1143881)
Supplement: Supplementary file 1 [file DataSheet_1.pdf]

SUPPLEMENTARY DATA

Supplementary Materials and Methods

Reagents and antibodies.

RPMI 1640, fetal calf serum (FCS), penicillin, trypsin and streptomycin were purchased from GIBCO (Massachusetts, USA). The Ficoll-Hypaque Plus used for cell separation was purchased from Amersham (Buckinhamshire, UK). BSA used for immunofluorescent staining buffer was obtained from Weiner Laboratories (Santa Fé, Argentina). Dimethyl sulfoxide (DMSO) was purchased from Sigma-Aldrich (Dallas, TX). Venetoclax (ABT-199), SKI-II and Opaganib were purchased from MedKoo Biosciences, Inc (Morrisville, NC, USA). PE- PECy5- or FITC- conjugated monoclal antibodies (mAbs) specific for human CD69 (clone FN50), CD38 (clone HB7), CD19 (clone HIB19), annexin-V FITC and propidium iodide (PI) were purchased from BD Bioscience, Pharmingen (CA, USA). FITC-, PE- or PerCP-Cy5-conjugated mAbs specific for human CD4 (clone OKT4), CD8 (clone HIT8a), PD-1 (clone NAT105), CD86 (clone 2331 FUN-1), CD40L (clone TRAP1), and CD49d (clone 9F10) and antibodies with irrelevant specificity (isotype controls) were obtained from BioLegend (CA, USA). Purified anti-CD3 (clone UCHT1) were obtained from Beckman Coulter (CA, USA). B-CLL human Microbeads isolation kit and Dead cell removal kit were obtained from Miltenyi biotec (Germany). RIPA buffer containing protease inhibitors were purchased from Thermo Fischer Scientific. Polyvinylidene difluoride (PVDF) membranes were purchased from Sigma-Aldrich (Dallas, TX). Rabbit mAb anti BCL-XL (clone 54H6), rabbit mAb anti-MCL-1 (clone D5V5L), rabbit mAb anti SPHK1 (clone D1H1L), rabbit mAb anti SPHK2 (clone D2V3G) and mouse mAb for β-actin (8H10D10) were purchased from Cell Signaling Tech (Massachusetts, USA). The HRP-conjugated mAb for mouse IgG was purchased from Sigma-Aldrich (USA) and the HRP-conjugated mAb for rabbit IgG from Jackson ImmunoResearch, Inc. and the enhanced chemiluminescence (ECL) kit used for visualized protein bands was purchased from Amersham.

CLL patients.

Peripheral blood samples were collected from twenty-eight unrelated CLL patients. All samples used in this study were obtained after informed consent in accordance with the Declaration of Helsinki and with Institutional Review Board approval from the Academia Nacional de Medicina, Buenos Aires, Argentina. CLL was diagnosed according to standard clinical and laboratory criteria. At the time of analysis, all patients were free from clinically relevant infectious complications and were treatment naïve or had not received treatment for ≥3 months before the investigation began. The main clinical and

biological characteristics of the patients enrolled in our study are summarized in **Supplementary Table 1**.

Cell separation procedures.

Peripheral blood mononuclear cells (PBMC) were isolated from fresh blood samples by density centrifugation over a Ficoll-Hypaque Plus gradient, washed twice with saline solution, and suspended in complete medium (RPMI 1640 supplemented with 10% FBS, 100 U/mL penicillin and 100 mg/mL streptomycin). Cells were used immediately or were cryopreserved in FCS 10% DMSO for further experiments.

Analysis of CD38 and CD49d expression by flow cytometry.

CD38 and CD49d expression on CLL cells was evaluated by flow cytometry (See **supplementary Table 1**). To this end, PBMC were stained with anti-CD38 PE mAb, anti-CD49d PE mAb or PE-conjugated isotype control Abs and PC5-conjugated mAb specific for CD19. Patients with ≥ 7 % of CLL cells expressing CD38 were considered $CD38^+$(1). On the other hand, patients with ≥ 30 % of CLL cells expressing CD49d were considered $CD49d^+$ (2).

Quantitation of viable cells.

Cell viability was evaluated by flow cytometric alterations of light-scattering properties (FSC-H), where apoptotic cells could be easily distinguished from viable cells because of their lower forward light scatter, consistent with reduction of cell size and cytoplasmic volume occurring during apoptosis. In these cases, cell survival was quantified as the percentage of cells within the gate of viable cells (3). These results were confirmed by Annexin V-FITC staining (not shown).

In vitro cultures and flow cytometry determinations

The effect of SPHK inhibitors on the generation of venetoclax resistance induced by activated T cells

PBMC from CLL patients ($4x10^6$ cells/ml) were cultured in complete medium with plate-bound anti-CD3 (aCD3 0,25 µg/ml, 96-well plate) or the isotype control (control cultures) in the presence of DMSO, SKI-II (15 µM) or opaganib (15 µM) for 48 hours and then venetoclax (0.2 µM) or DMSO (control) were added to the cultures for another 24 hours. The survival of $CD19^+$ cells was evaluated as mentioned above in control, venetoclax (Ven), aCD3 and aCD3 + venetoclax (aCD3+Ven) cultures of each treatment (DMSO, SKI-II and opaganib). With the aim to compare venetoclax-induced cell death in control

and in aCD3 cultures, these values were used to calculate the venetoclax resistance index for each patient as follows:

$$\text{Venetoclax Resistance Index} = \frac{\text{aCD3 + Ven}}{\text{aCD3}} \times \frac{\text{Control}}{\text{Ven}}$$

With this index, a value higher than 1 indicates that aCD3 cultures favor venetoclax resistance.

The effect of SPHK inhibitors on CLL and T cell activation

To evaluate whether SKI-II or opaganib affected the upregulation of CD86, PD-1 and PDL-1 on CLL cells induced by activated T cells, PBMC from CLL patients ($4x10^6$ cells/ml) were cultured in complete medium with plate-bound anti-CD3 (aCD3 0,25 µg/ml, 96-well plate) or the isotype control (control cultures) in the presence of DMSO, SKI-II (15 µM) or opaganib (15 µM) for 48 hours. The viability of $CD19^+$ cells was evaluated as previously mentioned. The characteristic of CLL cells within the gate of viable cells were studied by flow cytometry using anti-CD86-PE, anti-PD1-FITC, anti-PDL1-PE and anti-CD19-PECy5 monoclonal antibodies.

To evaluate whether SKI-II and opaganib affected the upregulation of CD40L and CD69 in T cells from CLL patients, PBMC from CLL patients ($4x10^6$ cells/ml) were cultured in complete medium with aCD3 or the isotype control in presence of DMSO, SKI-II (15 µM) or opaganib (15 µM). For CD40L determination, anti-CD40L-FITC mAb and monensin (2 µM), were added to the plate well from the beginning of the culture. The expression of CD40L was evaluated by flow cytometry on $CD4^+$ T cells at 24 hours and the expression of CD69 was evaluated on $CD4^+$ T cells and $CD8^+$ T cells at 24 hours.

The effect of SPHK inhibitors on the survival of venetoclax resistant cells

We first generated venetoclax resistant cells and then cultured these cells with or without SPHK inhibitors and venetoclax. To this aim, PBMC from CLL patients ($4x10^6$ cells/ml) were cultured in complete medium with aCD3 or the isotype control for 72 h in the presence of DMSO or venetoclax (0,2 µM) during the last 24 hours of culture. PBMC from control, aCD3 and aCD3+Ven cultures were washed and cultured with DMSO, SKI-II (15 µM) or opaganib (15 µM) for 96 hours combined with DMSO or venetoclax (0,2 µM) during the last 24 hours of culture. The survival of the cells was evaluated by flow cytometry as mentioned above.

Western blot analysis.

In order to analyze the expression of SPHK1 and SPHK2 on venetoclax resistant $CD19^{+}$ cells, PBMC from CLL patients ($4x10^{6}$ cells/ml) were cultured in complete medium with aCD3 or the isotype control. After 48 h, venetoclax 0,2 µM or DMSO were added to the cultures. After 24 h, we performed a two-step purification. First, we purified viable cells from control, aCD3 and aCD3 plus venetoclax cultures using Dead Cell Removal MicroBeads according to the manufacturer's instructions. Viable cells were always more than 95%. Then, leukemic B cells were obtained by negative selection with the anti-B-CLL Microbead isolation kit (purity obtained > 98%). Finally, whole-cell lysates were obtained from 3×10^{6} purified viable CLL cells using 26 µl of RIPA buffer containing protease inhibitors. Lysates were vortexed and incubated on ice twice, and then after centrifugation, supernatants were transferred to a new tube. 9 µl of loading buffer 4X containing 5% β-mercaptoethanol was added and then samples were incubated for 5 min at 95°. 30 µL of the protein extracts were separated on a standard 15% SDS-PAGE and transferred to PVDF membranes. Membranes were probed with primary antibodies for SPHK1, SPHK2 and β-Actin, followed by the corresponding secondary antibody. Specific bands were developed by enhanced chemiluminescence (ECL). Densitometric measurements of the specific bands were normalized to β-actin by using ImageJ (NIH).

To analyze whether the SPHK inhibitors affected the expression of BCL-XL and MCL-1 induced by autologous activated T cells, PBMC from CLL patients ($4x10^{6}$ cells/ml) were cultured in complete medium with plate-bound anti-CD3 (aCD3 0,25ug/ml, 6-well plate) or the isotype control (control cultures) in the presence of DMSO, SKI-II 15uM or opaganib 15uM. After 48 h, viable leukemic cells were isolated and western blot analysis was performed as described above with primary antibodies for BCL-XL, MCL-1 and β-Actin, followed by the corresponding secondary antibody.

Statistical analysis.

Statistical significance was determined using the nonparametric tests: Mann-Whitney test, Column Statistics followed by Wilcoxon's signed rank test, Wilcoxon matched-pairs test, Friedman test followed by Dunn's multiple comparison test. In all cases, $p<0.05$ was considered statistically significant. Data were analyzed using GraphPad Prism software version 7.00.

Supplementary table 1

Clinical and biological features of CLL patients enrolled in our study														
CLL#	Age (years)	Gender	Binet	Leucocytes, x10^{3}µl	Lymphocytes, x10^{3}µl	CD19$^+$% *	CD38$^+$ % ¥	CD49d$^+$% §	Platelets, x10^{3}µl	β2micro, µg/ml	LDH, U/l	HGB, g/dl	IGVH ¤	treatment
1	56	M	NA	NA	NA	85	35	90	NA	NA	NA	NA	U	no
2	81	F	A	89	81	89	35	24	257	NA	430	14.0	M	no
3	74	M	B	88	82,72	95	0,1	2	NA	NA	364	12	M	no
4	58	M	A	54	47,6	97,5	91	0,47	157	NA	113	15	U	no
5	61	M	B	170	167	73	3	1	169	7,7	225	12,4	U	NA
6	94	M	A	44,8	40,32	94,5	0,4	0,8	161	4,7	144	11,4	NA	NA
7	40	F	C	278	266,88	63	1	1	178	2,46	288	9,8	M	NA
8	67	F	A	NA	NA	63	17	38	NA	NA	NA	NA	NA	no
9	79	M	A	22.9	17,86	68	84	99	87	NA	338	11.5	U	no
10	NA	M	NA	NA	NA	84	2	1	NA	NA	NA	NA	NA	NA
11	71	F	A	46	41,6	81	1	93	187	3.6	119	11	M	no
12	85	M	C	650	539	97	10	1.30	102	NA	424	7,8	U	BR
13	72	F	C	92,78	65	87	16	0,4	110	5,1	511	12,3	NA	FCR, BO
14	55	F	C	71,57	64,43	86,1	2,22	0,77	111	3,09	NA	11	U	no
15	71	M	B	137	128,78	92,4	1,76	76,6	106	NA	NA	10.5	U	no
16	83	F	NA	NA	NA	92	0,33	49,4	120	NA	143	7,6	M	NA
17	74	F	B	113	91	94.6	5.04	1.62	199,2	2	480	13	NA	no
18	72	F	C	117	114,66	97,6	27,7	64,8	35	5,3	427	7,2	M	no
19	75	M	C	41,5	38,18	79	1	2,47	NA	3,5	146	NA	M	NA
21	82	M	B	59,6	53,64	97	0	0,4	171	4,5	170	11,8	U	BR
22	63	M	A	126	114,3	94	22	97	157	NA	171	13	M	I
23	71	M	C	38	31,3	95	1	4	157	3.1	160	10.8	U	no
24	77	M	B	39,2	36,456	93	8	1	152	2,5	198	14,2	M	no
25	73	M	B	114,9	110	85	44	6	282	1,9	385	14	M	no
26	62	M	C	188	153,596	95,4	0,3	0,3	284	3,4	345	NA	M	I
27	78	M	A	19,32	11	80.6	3.73	0.96	166	NA	NA	14.6	U	BR
28	49	M	C	360	342	92	82	95	34	5,43	235	8,4	U	no

M indicates male; **F**, female; **NA**, not available; **β2micro**, beta-2 microglobulin; **LDH**, lactate dehydrogenase; **HGB**, hemoglobin; **I**, ibrutinib; **R**, rituximab; **B**, bendamustine; **F**, fludarabine; **C**, cyclophosphamide; **O**, ofatumumab

£ Percentage of CD3$^+$ CD19$^-$ cells in peripheral blood lymphocytes

***** Percentage of CD19$^+$ cells (B cells, more than 99% CLL cells) in peripheral blood lymphocytes

¥ Percentage of CD38$^+$ cells in CD19$^+$ lymphocytes

§ Percentage of CD49d$^+$ cells in CD19$^+$ lymphocytes

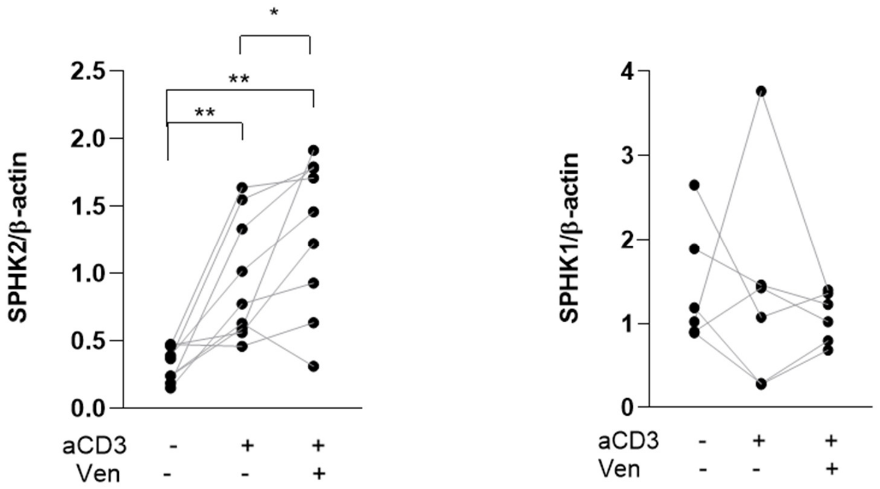

Supplementary Figure 1: Non-normalized data of the expression of SPHK1 and 2 in control and aCD3 cultures with or without venetoclax.

PBMC from CLL patients (4x106 cells/ml) were cultured in complete medium with aCD3 or the isotype control. After 48 h, venetoclax (Ven) 0,2 µM or DMSO were added to the cultures. After 24 h, non-viable cells were excluded by employing a dead cell removal kit and then CLL cells were purified using a CLL purification kit as detailed in the supplementary materials and methods section. Then, whole cell lysates were prepared with purified viable CLL cells and proteins were separated on a standard 12% SDS-PAGE and transferred to a PVDF membrane. Membranes were probed with primary antibodies for SPHK1, SPHK2 and β-Actin, followed by the corresponding secondary antibody. Then, specific bands were quantified by employing ImageJ and quantitative densitometry protein expression relative to β-actin as loading control was obtained for each culture condition. The figures show the expression of SPHK1 (n=6) and SPHK2 (n=9) relative to the expression of β-Actin. Statistical analysis was performed using Holm-Sidak's multiple comparisons test, * $p<0.05$, ** $p<0.01$.

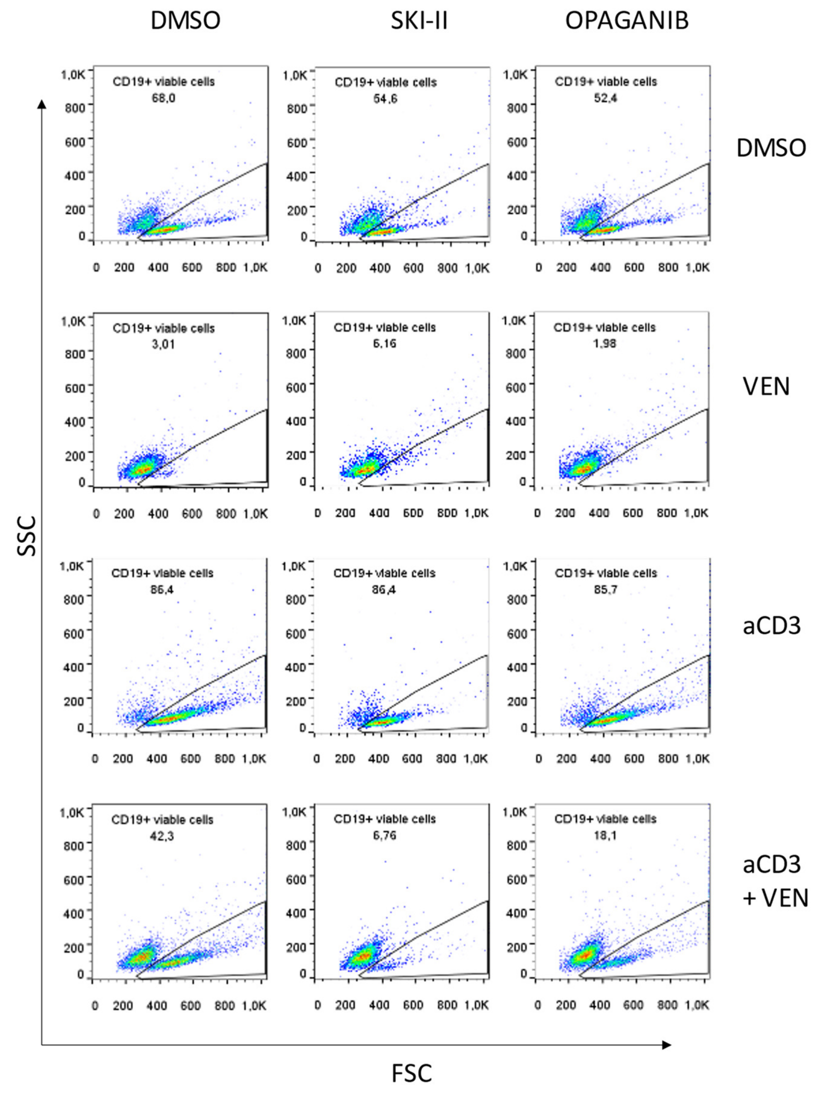

Supplementary Figure 2. SPHK inhibitors impair the generation of venetoclax resistance in CLL cells induced by activated T cells.

PBMC from CLL patients (4x10^6 cells/ml) were cultured in complete medium with aCD3 or the isotype control in presence or absence of SKI-II 15 µM and opaganib 15 µM for 48 h. Then, venetoclax 100 nM or DMSO were added to the cultures for additional 24 h. Cell viability of $CD19^+$ cells was evaluated by flow cytometric alterations of light-scattering properties (FSC-H), where apoptotic cells could be easily distinguished from viable cells because of their lower forward light scatter. The percentage of viable cells in each culture condition of one representative CLL sample (CLL#11) is shown.

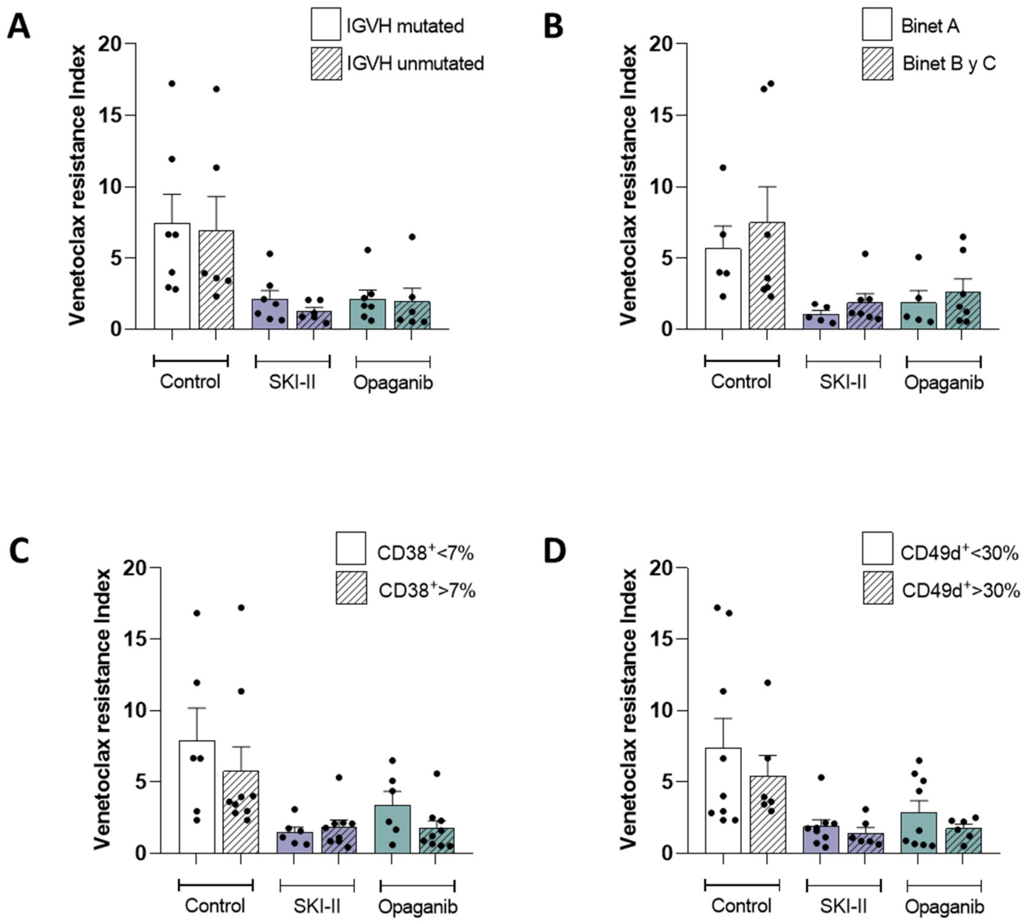

Supplementary Figure 3. Venetoclax resistance indexes are not associated with the clinical stage of the patients, their mutational status, or the expression of CD38 and CD49d.

PBMC from CLL patients ($4x10^6$ cells/ml) were cultured in complete medium with aCD3 or the isotype control in presence or absence of SKI-II (15 µM) and opaganib (15 µM) for 48 h. Then, venetoclax (Ven) 0,2 µM or DMSO were added to the cultures for additional 24 h. $CD19^+$ cell survival was evaluated by flow cytometry as detailed above. With the values of $CD19^+$ cell survival obtained in control, venetoclax (Ven), aCD3 and aCD3+Ven cultures we calculated the venetoclax resistance index for each patient as follows: (aCD3+Ven/aCD3) x (control/Ven). A value higher than 1 indicates that aCD3 cultures favor venetoclax resistance. **(A)** The figure shows the mean ± SEM of venetoclax resistance index of CLL patients (n=13) segregated in two groups based on their mutational status: IGVH mutated versus IGVH unmutated. **(B)** The figure shows the mean ± SEM of venetoclax resistance index of CLL patients (n=12) segregated in two

groups (BINET A versus BINET B-C. **(C)** The figure shows the mean ± SEM of venetoclax resistance index of CLL patients (n=15) segregated in two groups based on CD38 expression (CD38 negative versus CD38 positive). **(D)** The figure shows the mean ± SEM of venetoclax resistance index of CLL patients (n=15) segregated in two groups based on CD49d expression (CD49d negative versus CD49d positive). Statistical analysis to compare the two group of patients in each case was performed using Kruskall Wallis test followed by Dunn post-test.

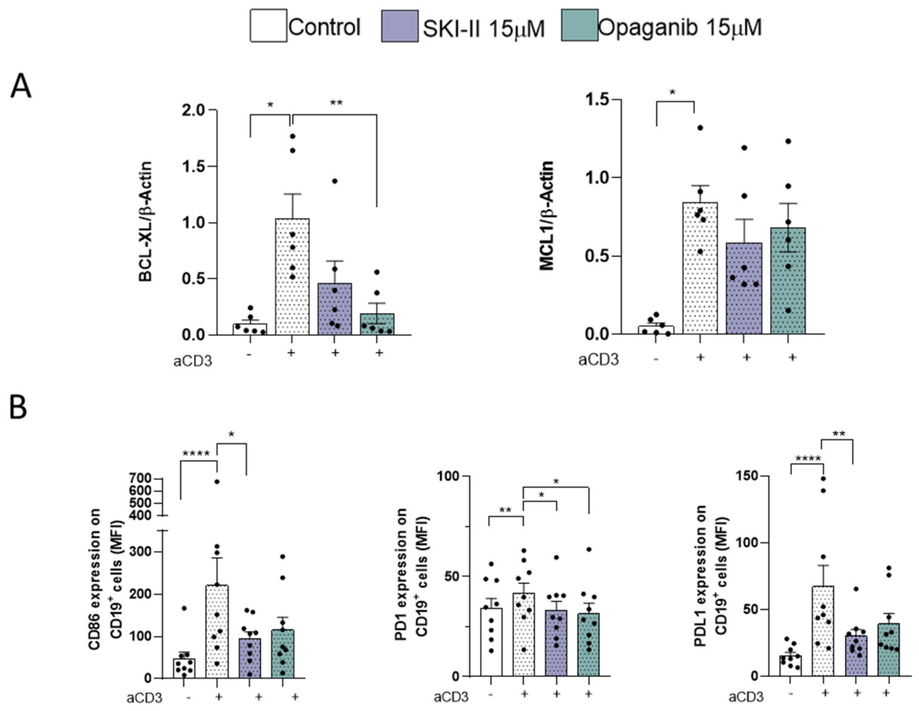

Supplementary Figure 4: Non-normalized data of the expression of BCL-XL, MCL-1 and activation markers in CLL cells from aCD3 cultures with or without SPHK inhibitors.

PBMC from CLL patients ($4x10^6$ cells/ml) were cultured in complete medium with aCD3 or the isotype control in presence or absence of SKI-II 15 µM and opaganib 15 µM for 48 h. **(A)** Purified CLL cells from control and aCD3 cultures with and without SKI-II and opaganib at 48 h were analyzed by western blot and membranes were probed with primary antibodies for BCL-XL, MCL-1 and β-Actin, followed by the corresponding secondary antibody. The figures show the expression of MCL-1 and BCL-XL on leukemic cells from control and aCD3 cultures relative to β-Actin. Statistical analysis was performed using Friedman test, * $p<0.05$, ** $p<0.01$ (n=6). **(B)** The expression of CD86, PD1 and PDL1 on $CD19^+$ cells was evaluated by flow cytometry at 48 h. The figures show CD86, PD1 and PDL1 expression on $CD19^+$ cells in control and aCD3 cultures. Statistical analysis was performed using Friedman test, * $p<0.05$, ** $p<0.01$, **** $p<0.0001$ (n=9).

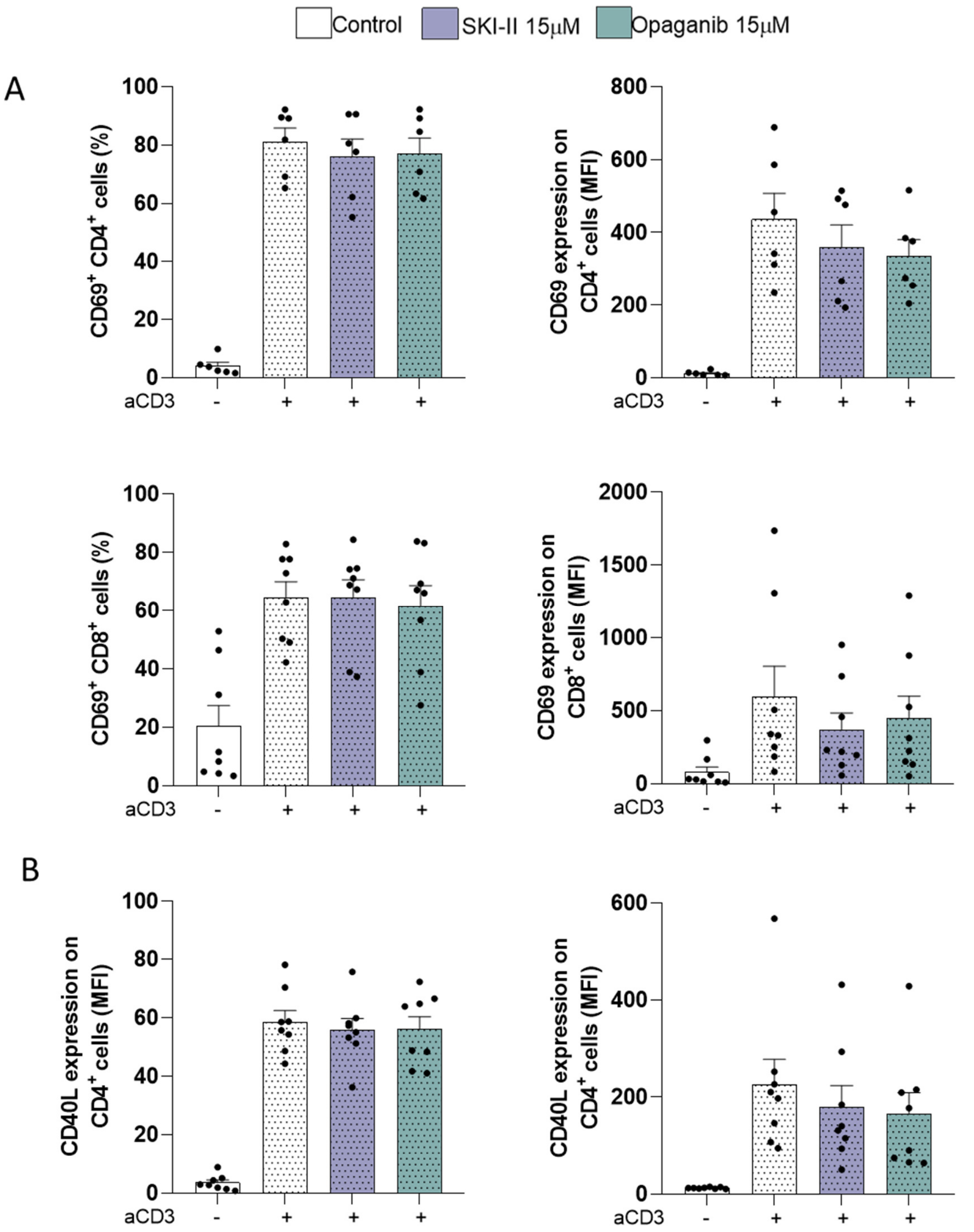

Supplementary Figure 5: Non-normalized data of the expression of CD69 and CD40L on T cells from CLL patients under SPHK inhibitors treatment.

PBMC from CLL patients ($4x10^6$ cells/ml) were cultured in complete medium with aCD3 or the isotype control in presence or absence of SKI-II 15 µM and opaganib 15 µM. **(A)**

The expression of CD69 on $CD4^+$ (n=6) and $CD8^+$ cells (n=8) were evaluated by flow cytometry at 24 h. In left panels, figures show the percentage of $CD69^+$ $CD4^+$ and $CD69^+$ $CD8^+$ cells; in right panels figures show the mean ± SEM of CD69 expression on $CD4^+$ and $CD69^+$ $CD8^+$ cells. **(B)** The expression of CD40L on $CD4^+$ cells was evaluated by flow cytometry at 24 h as detailed in supplementary methods (n=8). The figure shows the mean ± SEM of the percentage of $CD40L^+$ $CD4^+$ cells and the expression of CD40L on $CD4^+$ cells.

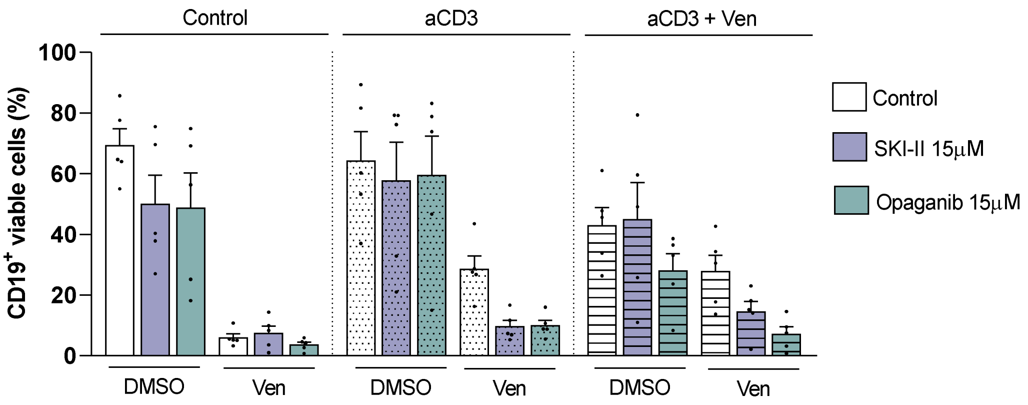

Supplementary Figure 6. Non-normalized data of the effect of SPHK inhibitors and venetoclax on the survival of resistant CLL cells.

PBMC from CLL patients (4x10^6 cells/ml) were cultured in complete medium with aCD3 or the isotype control for 72 h in the presence of DMSO or venetoclax (Ven) during the last 24 h of culture. Then, PBMC from control cultures, aCD3 cultures and aCD3+VEN cultures were washed and cultured with DMSO, SKI-II 15 µM or opaganib 15 µM for 96 h combined with DMSO or venetoclax (Ven) during the last 24 h of culture. The figure shows the values of non-normalized data of the cell survival in each condition that were employed for Figure 4 C-D (n= 5).

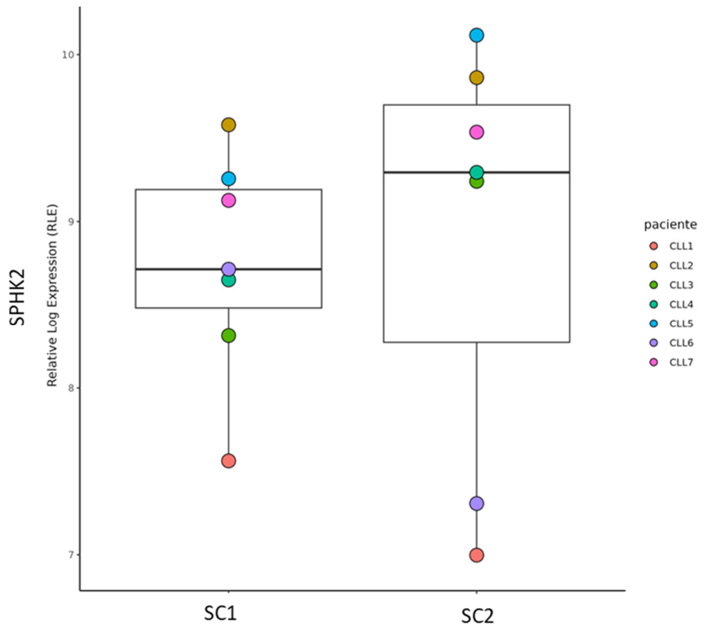

Supplementary Figure 7. SPHK2 expression in Series GSE192685 (4) pre venetoclax treatment (SC1) and post persistent MRD after 1 year on venetoclax (SC2).

1. Gentile M, Mauro FR, Calabrese E, De Propris MS, Giammartini E, Mancini F, et al. The prognostic value of CD38 expression in chronic lymphocytic leukaemia patients studied prospectively at diagnosis: a single institute experience. Br J Haematol. 2005;130(4):549-57.
2. Bulian P, Shanafelt TD, Fegan C, Zucchetto A, Cro L, Nuckel H, et al. CD49d is the strongest flow cytometry-based predictor of overall survival in chronic lymphocytic leukemia. Journal of clinical oncology : official journal of the American Society of Clinical Oncology. 2014;32(9):897-904.
3. Morande PE, Zanetti SR, Borge M, Nannini P, Jancic C, Bezares RF, et al. The cytotoxic activity of Aplidin in chronic lymphocytic leukemia (CLL) is mediated by a direct effect on leukemic cells and an indirect effect on monocyte-derived cells. Investigational new drugs. 2012;30(5):1830-40.
4. Ghia EM, Rassenti LZ, Choi MY, Quijada-Alamo M, Chu E, Widhopf GF, 2nd, et al. High expression level of ROR1 and ROR1-signaling associates with venetoclax resistance in chronic lymphocytic leukemia. Leukemia. 2022;36(6):1609-18.
